# School Vaccine Coverage and Medical Exemption Uptake After the New York State Repeal of Nonmedical Vaccination Exemptions

**DOI:** 10.1001/jamanetworkopen.2023.54710

**Published:** 2024-02-02

**Authors:** John W. Correira, Rhiannon Kamstra, Nanqing Zhu, Margaret K. Doll

**Affiliations:** 1 Department of Allied Health Sciences, Albany College of Pharmacy and Health Sciences, Albany, New York; 2 Precision Analytics, Montreal, Quebec, Canada

## Abstract

**Importance:**

Although New York State (NYS) recently adopted legislation eliminating nonmedical vaccination exemption options from school-entry requirements, the implications of the law for school vaccine coverage and medical vaccine exemption uptake have not been examined.

**Objective:**

To evaluate the implications of the repeal of school-entry nonmedical vaccination exemptions for vaccine coverage and medical exemption uptake at NYS schools outside of New York City (NYC).

**Design, Setting, and Participants:**

This cohort study had an interrupted time-series design and used generalized estimating equation models to examine longitudinal school immunization compliance data from the 2012 to 2013 through 2021 to 2022 school years. The cohort comprised NYS public and nonpublic schools, excluding NYC schools, with any students enrolled in kindergarten to 12th grade. Eligible schools had enrollment and immunization data before and after the implementation of the Senate Bill 2994A legislation. Data analyses were conducted in July 2023.

**Exposure:**

Senate Bill 2994A was passed in June 2019, eliminating school-entry nonmedical vaccination exemptions. Since compliance with the law was evaluated for most students during the next school year, the 2019 to 2020 school year was considered to be the law’s effective date.

**Main Outcomes and Measures:**

The primary outcomes were school vaccine coverage (defined as the percentage of students at each school who completed grade-appropriate requirements for all required vaccines) and medical exemption uptake (defined as the percentage of students at each school who received a medical exemption).

**Results:**

Among the 3821 eligible schools, 3632 (95.1%) were included in the analysis, representing 2794 (96.9% of eligible) public schools and 838 (89.2% of eligible) nonpublic schools. The implementation of Senate Bill 2994A was associated with absolute increases in mean vaccine coverage of 5.5% (95% CI, 4.5%-6.6%) among nonpublic schools and 0.9% (95% CI, 0.7%-1.1%) among public schools, with additional annual increases in vaccine coverage observed through the 2021 to 2022 school year. The law’s implementation was also associated with a 0.1% (95% CI, 0.0%-0.1%) mean absolute decrease in medical vaccination exemption uptake at both public and nonpublic schools, and small but significant mean annual decreases in medical vaccination exemptions (0.02%; 95% CI, 0.01%-0.03%) through the end of the study period.

**Conclusions and Relevance:**

Results of this cohort study suggested that repeal of school-entry nonmedical vaccination exemptions was associated with increased vaccine coverage at NYS schools outside of NYC. Coverage gains were not replaced by increases in medical vaccination exemptions.

## Key Points

**Question:**

Was the New York State (NYS) law repealing nonmedical vaccination exemption options from school-entry requirements (Senate Bill 2994A) associated with an increase in vaccine coverage in NYS schools outside of New York City?

**Findings:**

In this cohort study of 3632 schools, Senate Bill 2994A was associated with an increase in mean vaccine coverage at NYS schools. Small but significant decreases in medical exemptions were also observed.

**Meaning:**

Findings of this study suggest that state legislation eliminating nonmedical vaccination exemptions from school-entry requirements can be effective in increasing school vaccine coverage without replacement by medical vaccination exemptions.

## Teaser

This cohort study examines the completion of required vaccinations and medical exemption use among kindergarten to 12th grade students before and after the passage of a law repealing nonmedical vaccination exemptions in New York State.

## Introduction

After 2 large measles outbreaks in New York State (NYS) that threatened the US measles elimination status, the NYS legislature passed Senate Bill 2994A in June 2019, repealing nonmedical vaccination exemption options from school-entry immunization requirements.^1,2,3^ With the law’s passage, New York became the fifth US state to address increasing pediatric undervaccination by eliminating nonmedical vaccination exemptions at schools and the third US state, behind California and Maine, to adopt such a law in recent decades.^4,5,6,7^ Since Senate Bill 2994A was enacted, other states have considered similar legislation, including Connecticut, where nonmedical vaccination exemptions were repealed in 2021.^8^ Yet, unlike recent laws passed in California, Maine, and Connecticut, Senate Bill 2994A became effective immediately and did not include a grandfather clause excusing students with existing nonmedical exemptions from compliance.^2,6,7,8^

While school-entry immunization requirements effectively promote and maintain high US pediatric vaccine coverage,^9,10^ less is known regarding the outcome of legislative repeals of nonmedical vaccination exemption options. Initial evaluations of a California repeal of school nonmedical vaccination exemptions suggest that although the legislation was effective to increase the percentage of kindergartners up-to-date for vaccinations, these increases were partially offset by simultaneous increases in student medical vaccine exemptions.^4,7,11,12^ In addition, new medical vaccination exemptions were found to spatially cluster, suggesting that the legislation did not have a uniform impact on vaccine compliance.^4,13^

To our knowledge, to date, the results of the NYS vaccination exemption repeal have not been evaluated. Furthermore, given the differences between state laws, it is feasible that outcomes of similar legislation may differ by jurisdiction. In this cohort study, we aimed to evaluate the implications of the NYS repeal of school-entry nonmedical vaccination exemptions for vaccine coverage and medical exemption uptake at NYS schools outside of New York City (NYC).

## Methods

### Study Setting and Design

Senate Bill 2994A was signed into law on June 13, 2019.^2^ Although this law became effective immediately, students were given a 14-day grace period to demonstrate compliance or a pathway toward compliance (ie, receipt of the first vaccination for all required vaccinations) prior to exclusion from NYS schools.^2^ Due to the timing of this grace period and school summer recess, compliance for most students was not assessed until the 2019 to 2020 school year. We conducted this cohort study with interrupted time-series analyses using data from the 2012 to 2013 through 2021 to 2022 school years at schools that enrolled any students from kindergarten to 12th grade (K-12). For these analyses, we defined the 2019 to 2020 school year as the legislation’s effective date. In accordance with the Common Rule, this cohort study was exempt from ethics review and the informed consent requirement because it used only publicly available deidentified data. We followed the Strengthening the Reporting of Observational Studies in Epidemiology (STROBE) reporting guideline.^14^

### Data Sources and Study Population

We used publicly available data from 2 NYS governmental sources: (1) the Education Department Information and Reporting Services to ascertain school enrollments during the study period, and (2) the Department of Health School Immunization Survey to identify school immunization status. Since NYC and non-NYC schools have different governance structures, publicly available data, and immunization registries, we excluded NYC schools from all analyses.

After exclusion of NYC schools, we created a cohort of NYS schools using the Information and Reporting Services school enrollment data for the 2012 to 2013 through 2021 to 2022 school years.^15^ Briefly, Information and Reporting Services is charged with collecting and reporting school data for NYS public schools, charter schools, and participating nonpublic schools. Annual enrollment reports are required for public and charter schools and for nonpublic schools that wish to access selected NYS resources.^16^ For the present study, we defined eligibility for inclusion as schools reporting enrollment of K-12 students both before (2012-2013 through 2018-2019 school years) and after (2019-2020 through 2021-2022 school years) the implementation of Senate Bill 2994A. Race and ethnicity data were analyzed to describe the demographic characteristics of students in eligible schools.

Next, we merged the Education Department Information and Reporting Services enrollment data from eligible schools with the Department of Health School Immunization Survey data.^17,18^ Briefly, this annual survey requires schools to report the number of K-12 students with medical and nonmedical vaccination exemptions and students who met grade-appropriate proof of immunity requirements for 7 mandatory school-entry immunizations.^17,19^ The Department of Health publicly releases aggregated, annual survey data for each school reporting the percentages of students for each variable. The Education Department and Department of Health school data were merged using the school’s unique Basic Education Data System identifier (BEDS ID); however, discrepancies were noted for some BEDS IDs in the Department of Health files. To ensure appropriate linkages, 2 of us (J.W.C., M.K.D.) manually reviewed school names and location information from both the Education Department and Department of Health datasets for all BEDS IDs. Where school names and/or locations were discrepant between datasets or were unmerged, efforts were made to match Department of Health reports to other BEDS IDs appearing in the Education Department datasets. The BEDS IDs for schools that could not be confirmed were coded as missing. To be included in analyses, eligible schools must have submitted to the Department of Health immunization data in both periods before and after Senate Bill 2994A passage.

### Statistical Analysis

In separate analyses, we investigated the role of Senate Bill 2994A in the following primary outcomes: (1) vaccine coverage, defined as the annual percentage of students at each school who completed grade-appropriate requirements for all 7 required vaccines, and (2) medical exemption uptake, defined as the percentage of students at each school who received a medical exemption. In crude analyses, we explored these outcomes as both (1) unweighted estimates, where schools contributed equally to inform coverage across school clusters, and (2) weighted estimates, where outcome data were weighted by a school’s enrollment to inform coverage across student populations. In crude analyses, we compared outcomes by school type and year using absolute mean differences and 95% CIs; mean differences with 95% CIs that did not cross 0% were regarded as statistically significant.

In adjusted analyses that accounted for repeated sampling of schools, we used generalized estimating equation (GEE) models with a binomial distribution, identity link function, and first-order autoregressive structure to estimate the implications of the law for each of the primary outcomes as absolute mean differences and 95% CIs. In these analyses, the law was modeled as a binary variable, representing 0 for the period before and 1 for the period after the law’s implementation. Because we anticipated that Senate Bill 2994A results may change over time, we considered adjustment for a change in trend in the postimplementation period using a product term between the binary law variable and a linear variable, accounting for the school year after passage of the law. Additionally, since we anticipated that the law’s implications may differ by school type,^20,21^ we considered inclusion of a binary variable, accounting for nonpublic and public school types and separate product terms for differences in trend by school type in both pre- and postimplementation periods. Each of these product terms was retained in GEE models if the variable was significant in the fully adjusted model (2-sided *P* < .05). In secondary analyses, we examined the percentage of students with any (ie, nonmedical and medical) vaccination exemptions as the outcome of interest and followed similar model-fitting procedures.

All analyses were conducted in July 2023 using RStudio with R, version 4.2.2 (R Project for Statistical Computing). The geepack R package, version 1.3.9, was used to implement GEE models.

## Results

### Included and Excluded Schools 

**Figure 1.**
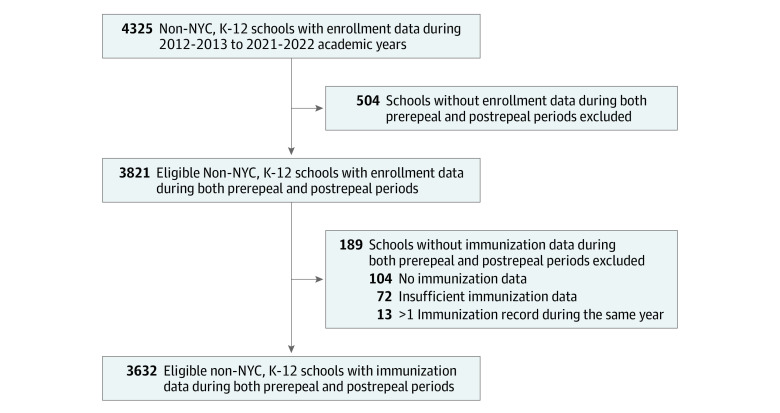
Flow Diagram of Included Schools K-12 indicates kindergarten through 12th grade; NYC, New York City.

Figure 1 depicts a flow diagram of NYS schools considered for study inclusion. Briefly, we identified 4325 NYS schools outside of NYC with K-12 enrollment data for the 2012 to 2013 through 2021 to 2022 academic years. Of these schools, 3821 (2882 public and 939 nonpublic) had enrollment data in both preimplementation and postimplementation periods, representing eligible schools. After merging eligible schools with NYS Department of Health immunization data, we identified 3632 schools (95.1%) with sufficient immunization data and included them in the analyses, representing 2794 (96.9% of eligible) public schools and 838 (89.2% of eligible) nonpublic schools. Across the 10-year study period, these schools submitted 34 784 immunization reports, with a mean of 9.8 (95% CI, 9.8-9.8) reports among public schools and 8.9 (95% CI, 8.7-9.0) reports among nonpublic schools. When only school years with enrollment data were considered, 3525 included schools (97.1%) submitted complete student enrollment and immunization data; an additional 79 schools (2.2%) were missing immunization data for only 1 school year (eTable 1 in Supplement 1).

Characteristics of eligible included (n = 3632) and excluded (n = 189) schools are provided in eTable 2 in Supplement 1. Excluded schools were less likely to be public (46.6% [95% CI, 39.4%-53.7%] vs 76.9% [95% CI, 75.6%-78.3%]), have a smaller student enrollment (264.7 [95% CI, 216.7-312.8] vs 457.3 [95% CI, 445.0-469.6]), and a higher percentage of students who identified as having White race and ethnicity (78.7% [95% CI, 74.8%-82.6%] vs 65.8% [95% CI, 64.8%-66.8%]) compared with included schools.

### Vaccine Coverage

The Table presents crude analyses of mean vaccine coverage. During the 2012 to 2013 school year, overall mean coverage weighted by student enrollment was 98.4% (95% CI, 98.2%-98.6%) and decreased to 96.8% (95% CI, 96.5%-97.1%) in 2018 to 2019, the school year prior to Senate Bill 2994A implementation. During this same period, unweighted mean coverage started at 96.5% (95% CI, 96.1%-96.9%) and decreased to 94.5% (95% CI, 94.1%-94.9%). In both weighted and unweighted mean estimates, vaccine coverage was consistently lower among nonpublic schools during the preimplementation period. In the year before Senate Bill 2994A passage, weighted mean coverage was 97.6% (95% CI, 97.5%-97.8%) among public school students and 88.4% (95% CI, 85.3%-91.4%) among nonpublic school students, while unweighted mean coverage was 97.6% (95% CI, 97.5%-97.7%) at public schools and 84.1% (95% CI, 82.5%-85.8%) at nonpublic schools. After the law’s implementation, weighted mean vaccine coverage increased by 0.8% (95% CI, 0.6%-0.9%) among public school students and 5.8% (95% CI, 2.5%-9.0%) among nonpublic school students, while unweighted mean coverage increased by 0.7% (95% CI, 0.5%-0.9%) at public schools and 5.0% (95% CI, 2.7%-7.2%) at nonpublic schools. Small annual increases in vaccine coverage were also observed through the 2021 to 2022 school year, with weighted mean vaccine coverage of 98.4% (95% CI, 98.3%-98.5%) among public school students and 95.2% (95% CI, 94.2%-96.3%) among nonpublic school students and with unweighted mean estimates of 98.5% (95% CI, 98.4%-98.6%) at public schools and 89.9% (95% CI, 88.1%-91.7%) at nonpublic schools. At the conclusion of the study period, public schools had 3.2% (95% CI, 2.1%-4.2%) higher weighted mean coverage and 8.6% (95% CI, 6.8%-10.4%) higher unweighted mean coverage compared with nonpublic schools.

**Table.** Vaccine Coverage at New York State Schools Outside of New York City Before and After the 2019 Passage of Senate Bill 2994A

Start of academic year	Unweighted mean school vaccination coverage, % (95% CI)			Weighted mean student vaccination coverage, % (95% CI)		
	Overall	Public	Nonpublic	Overall	Public	Nonpublic
Pre–Senate Bill 2994A						
2012	96.5 (96.1-96.9)	98.6 (98.5-98.7)	88.1 (86.3-89.8)	98.4 (98.2-98.6)	98.7 (98.5-98.9)	95.3 (94.4-96.1)
2013	96.4 (96.0-96.8)	98.6 (98.5-98.7)	87.9 (86.2-89.6)	98.3 (98.2-98.4)	98.6 (98.5-98.8)	94.7 (93.8-95.6)
2014	95.5 (95.1-95.9)	97.8 (97.7-97.9)	86.8 (85.1-88.5)	97.6 (97.4-97.7)	97.9 (97.7-98.1)	93.8 (92.8-94.8)
2015	95.2 (94.8-95.6)	97.8 (97.7-97.9)	85.8 (84.0-87.5)	97.5 (97.3-97.7)	98.0 (97.8-98.1)	92.4 (91.0-93.8)
2016	95.0 (94.6-95.4)	97.7 (97.5-97.8)	85.5 (83.8-87.2)	97.0 (96.7-97.3)	97.5 (97.3-97.8)	91.9 (90.1-93.6)
2017	94.5 (94.0-94.9)	97.3 (97.2-97.5)	84.5 (82.8-86.2)	96.7 (96.4-97.0)	97.3 (97.1-97.6)	90.4 (88.6-92.2)
2018	94.5 (94.1-94.9)	97.6 (97.5-97.7)	84.1 (82.5-85.8)	96.8 (96.5-97.1)	97.6 (97.5-97.8)	88.4 (85.3-91.4)
Post–Senate Bill 2994A						
2019	96.2 (95.8-96.6)	98.3 (98.1-98.5)	89.1 (87.6-90.6)	98.0 (97.9-98.2)	98.4 (98.3-98.5)	94.1 (93.1-95.2)
2020	97.3 (96.9-97.6)	99.0 (98.8-99.1)	90.9 (89.4-92.5)	98.6 (98.4-98.9)	98.9 (98.7-99.2)	95.1 (94.2-96.0)
2021	96.9 (96.5-97.3)	98.5 (98.4-98.6)	89.9 (88.1-91.7)	98.2 (98.0-98.3)	98.4 (98.3-98.5)	95.2 (94.2-96.3)

**Figure 2.**
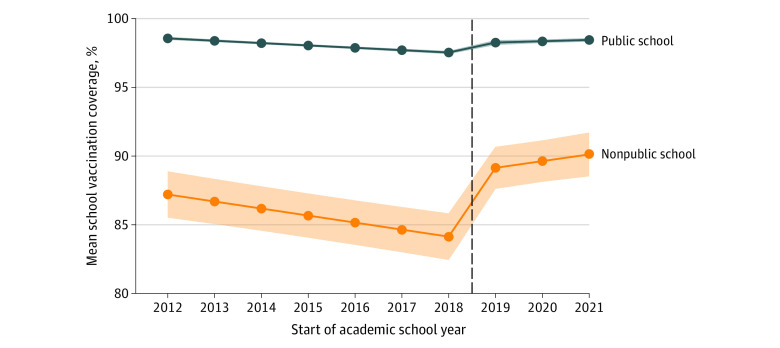
Generalized Estimating Equation–Modeled Estimates of School Vaccine Coverage by School Type The New York State 2019 Senate Bill 2994A (repeal of nonmedical exemptions) affected students in the 2019 to 2020 school year. The shaded area represents the 95% CIs. The dashed line represents implementation of the law.

Final selected GEE models examining school vaccine coverage contained school type terms and product terms to allow for differences in trend by school type in both preimplementation and postimplementation periods (eTable 3 in Supplement 1). Figure 2 displays modeled vaccine coverage estimates. In general, the trends were similar to those of the crude analyses. Briefly, baseline coverage was higher among public than nonpublic schools. While both school types experienced annual decreases in coverage during the preimplementation period, decreases in mean coverage among nonpublic schools were greater. After Senate Bill 2994A implementation, mean vaccine coverage increased by 0.9% (95% CI, 0.7%-1.1%) among public schools and 5.5% (95% CI, 4.5%-6.6%) among nonpublic schools. Both public and nonpublic schools also experienced annual increases of 0.3% (95% CI, 0.2%-0.4%) and 1.0% (95% CI, 0.6%-1.4%) in mean vaccination coverage, respectively, during the postimplementation period (eTable 3 in Supplement 1).

### Medical Exemption Uptake

**Figure 3.**
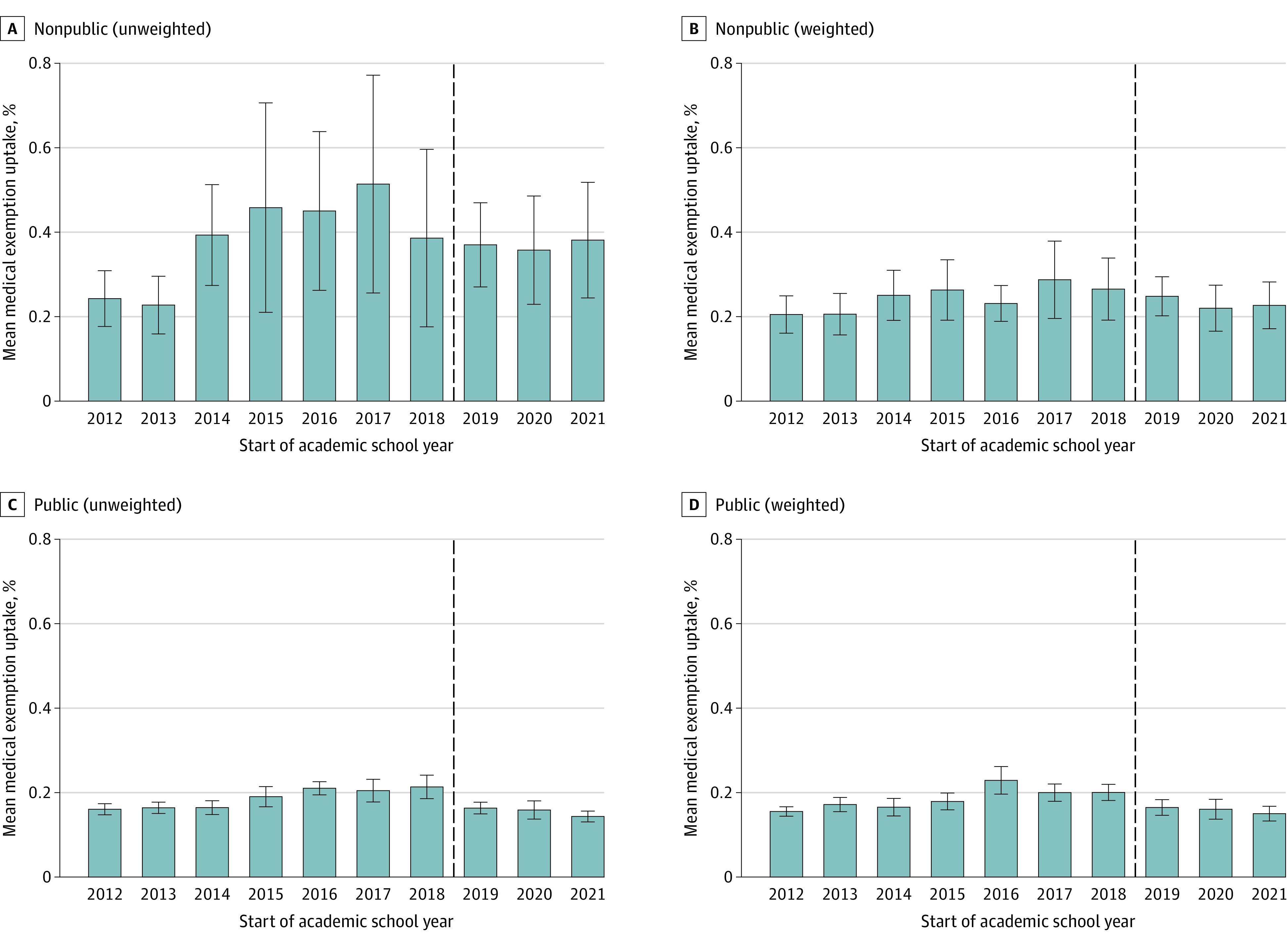
Uptake of Medical Vaccination Exemptions by School Type The New York State 2019 Senate Bill 2994A (repeal of nonmedical exemptions) affected students in the 2019 to 2020 school year. Error bars represent 95% CIs. Variables represent weighted and unweighted mean percentages with 95% CIs. The dashed line represents implementation of the law.

Figure 3 examines weighted and unweighted mean medical vaccination exemption uptake among public and nonpublic schools over time. In both weighted and unweighted analyses during the preimplementation period, medical exemptions were more common among nonpublic than public schools and increased slightly. However, medical exemptions were rare in the preimplementation period, accounting for a weighted mean of 0.2% (95% CI, 0.2%-0.2%) of public school students and 0.3% (95% CI, 0.2%-0.3%) of nonpublic school students. After the Senate Bill 2994A implementation, the weighted mean of medical exemptions among public and nonpublic school students remained unchanged. Final GEE models examining this outcome contained a term for school type and separate terms for differences in trend in preimplementation and postimplementation periods (eTable 4 in Supplement 1). Among public and nonpublic schools, a small but significant increase in medical exemptions was observed during the preimplementation period. In the school year after implementation, these schools experienced a 0.1% (95% CI, 0.0%-0.1%) decrease in medical exemptions, with annual declines of 0.02% (95% CI, 0.01%-0.03%) observed through the end of the study period.

### Uptake of Any Vaccination Exemptions

**Figure 4.**
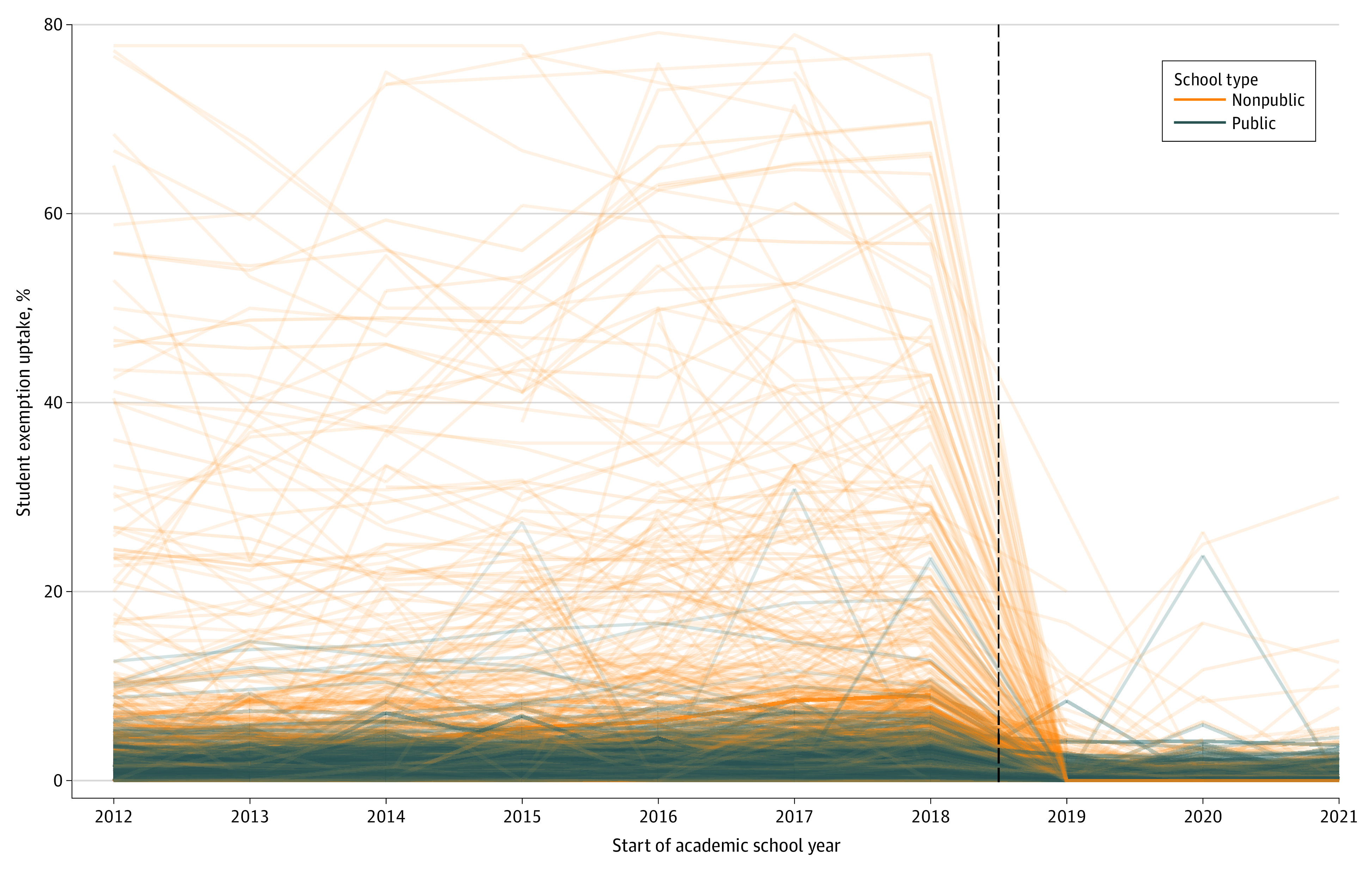
Uptake of School Vaccination Exemptions Over Time Among Public and Nonpublic Schools Each line represents data for an individual school, with line transparency weighted by student enrollment (ie, lower enrollment depicted as a more transparent line). The New York State 2019 Senate Bill 2994A (repeal of nonmedical exemptions) affected students in the 2019 to 2020 school year. Schools with vaccine exemption percentages above 80% are not displayed. The dashed line represents implementation of the law.

Figure 4 shows crude data for any vaccination exemption uptake over time by school type (ie, public or nonpublic). Overall, weighted estimates found that 1.0% (95% CI, 0.9%-1.0%) of public school students and 4.1% (95% CI, 3.5%-4.6%) of nonpublic school students reported a vaccination exemption in the year prior to Senate Bill 2994A implementation. Unweighted estimates showed a mean exemption uptake of 1.1% (95% CI, 1.0%-1.1%) among public schools and 9.9% (95% CI, 8.4%-11.3%) among nonpublic schools. After Senate Bill 2994A implementation, the mean exemption uptake decreased in both weighted and unweighted estimates. At the end of the study period (2021-2022), the weighted mean uptake of any vaccination exemptions was 0.2% (95% CI, 0.1%-0.2%) among public school students and 0.2% (95% CI, 0.2%-0.3%) among nonpublic school students, with unweighted estimates of 0.1% (95% CI, 0.1%-0.2%) among public and 0.4% (95% CI, 0.2%- 0.5%) among nonpublic schools. Final GEE models examining this outcome contained terms for school type and product terms to allow for differences in trend by school type in the preimplementation and postimplementation periods (eTable 5 in Supplement 1), yielding results similar to those from crude analyses. After the law’s implementation, public and nonpublic schools experienced 1.0% (95% CI, 1.0%-1.0%) and 9.7% (95% CI, 8.3%-11.2%) decreases in vaccination exemption uptake, respectively. Additional annual decreases of 0.1% (95% CI, 0.1%-0.1%) at public schools and 0.3% (95% CI, 0.1%-0.4%) at nonpublic schools were observed through the 2021 to 2022 school year.

## Discussion

In this cohort study, we observed consistent decreases in school vaccine coverage over time prior to Senate Bill 2994A implementation, with unweighted mean coverage of 84.1% among nonpublic schools and 97.6% among public schools in the school year prior to the law’s adoption. Using GEE models, we estimated that Senate Bill 2994A implementation was associated with absolute increases in mean vaccine coverage of 5.5% among nonpublic schools and 0.9% among public schools, with additional annual mean increases of 1.0% among nonpublic schools and 0.3% among public schools estimated through the 2021 to 2022 school year. Additionally, we found that Senate Bill 2994A implementation was associated with a small but significant absolute decrease of 0.1% in medical exemption uptake among nonpublic and public schools, with subsequent annual mean decreases of 0.02% among these schools through the 2021 to 2022 school year. Collectively, these results suggest that repeal of nonmedical vaccination exemptions was effective in addressing decreasing vaccine coverage at NYS schools outside of NYC and that coverage gains were not replaced by increases in medical vaccination exemptions.

To our knowledge, this study was the first to evaluate the implications of Senate Bill 2994A for vaccine coverage and medical exemption uptake. Although initial evaluations of a similar repeal in California (Senate Bill 277) suggest that decreases in nonmedical vaccination exemptions for schools were partially offset by the uptake of new medical exemptions,^4,7,11,12^ we found that NYS schools outside of NYC did not share these experiences. Instead, we hypothesized that the small but significant decreases in medical exemption uptake after Senate Bill 2994A passage in NYS may be associated with revisions of administrative regulations pertaining to medical exemptions, which were introduced shortly after the law was adopted.^22^ Specifically, these revisions clarified the definition of the term *medical exemption* and outlined a rigorous documentation process for students to obtain a medical exemption.^22^ Conversely, California’s 2015 Senate Bill 277 contained explicit provisions that broadened the use of medical exemptions to include reasons that did not represent contraindications to vaccination, such as a family medical history.^5,12^ Although California legislators subsequently amended these provisions in 2019,^23^ the apparent differences in initial experiences between California and NYS may have implications for policymakers in other jurisdictions designing similar legislation. Nonetheless, additional research on the longitudinal trends and spatial clustering of medical exemptions among NYS schools is warranted to ascertain whether these findings remain stable and are uniform across NYS geographic regions.

The crude analyses examining weighted vs unweighted mean vaccine coverage demonstrated the public health challenges of addressing undervaccination to prevent vaccine-preventable disease outbreaks in the population. While the weighted mean estimates showed high overall vaccine coverage of 96.8% among NYS school students in the year prior to Senate Bill 2994A implementation, unweighted mean vaccine coverage during the same period was only 84.1% at nonpublic schools. This contrast highlights the apparent clustering of vaccination exemptions among NYS nonpublic schools, which was previously described in both secular and nonsecular settings among subpopulations that may be less accepting of immunizations.^21^ Although this study presented evidence that Senate Bill 2994A was effective in increasing mean vaccine coverage, differences in vaccine coverage between public and nonpublic schools persisted, with public schools having 3.2% higher weighted mean vaccine coverage or 8.6% higher unweighted mean vaccine coverage in comparison with nonpublic schools at the end of the study period. These apparent differences have implications for the control and spread of vaccine-preventable diseases in the population and suggest greater susceptibility to vaccine-preventable disease outbreaks among nonpublic schools, where disease may be more readily introduced and spread among closed communities with insufficient vaccine coverage.^21^

### Limitations

This study has several limitations. First, the study period was limited to 3 school years after the Senate Bill 2994A implementation. Although this limitation was offset by the study design, which allowed us to consider long-term school vaccine coverage trends in the 7 years prior to the implementation of the law, monitoring the longitudinal implications of Senate Bill 2994A remains important to understanding its effectiveness in maintaining high levels of NYS school vaccine coverage. Second, the 3 school years we examined after Senate Bill 2994A implementation coincided with the COVID-19 pandemic. Where the pandemic prompted changes in school immunization enforcement and documentation, the internal or external validity of the findings may be affected. Nonetheless, the pandemic did not prompt NYS school closures until March 2020, making it less likely that changes in vaccine coverage observed for the 2019 to 2020 school year (ie, the first year after Senate Bill 2994A implementation) were affected by the pandemic. Third, the study analyses relied on publicly available datasets, which may not include nonpublic schools that did not want to access certain NYS services or reimbursement.^16^ Furthermore, due to reporting differences by jurisdiction, we excluded NYC schools; thus, the findings may not be generalizable to this region. Fourth, the study excluded approximately 5% of eligible schools due to incomplete immunization data, with differences in inclusion by school type (96.9% of public schools vs 89.2% of nonpublic schools). Should excluded schools be less likely to comply with immunization requirements, the analyses may overestimate the effectiveness of Senate Bill 2994A. Nonetheless, the analyses remain important as they represent the experiences of 95% of NYS schools outside of NYC. Fifth, we did not examine whether Senate Bill 2994A was associated with changes in school enrollment. Research in both California and NYS has suggested that students may leave traditional school systems to avoid compliance with strict immunization laws,^23,24^ which may have implications for the spread of vaccine-preventable diseases in the broader population, despite observed increases in school immunization coverage. Sixth, the study did not evaluate the percentage of students on a pathway to immunization compliance. These students may represent persons with insufficient immunity, who can also facilitate the spread of vaccine-preventable diseases.

## Conclusions

In this cohort study with interrupted time-series analyses, the repeal of nonmedical vaccination exemptions was associated with increases in mean vaccine coverage at NYS schools outside of NYC. Among these schools, evidence that coverage gains were offset by increasing medical exemptions was not found; instead, a small but significant decrease in medical exemptions associated with implementation of Senate Bill 2994A was observed. These findings suggest that a legislative repeal of school-entry nonmedical vaccination exemptions can be effective in increasing vaccination compliance without replacement by new medical exemptions. Nevertheless, continued research is necessary to examine spatial clustering of immunization exemptions, potential changes in student enrollment, and long-term effectiveness of Senate Bill 2994A in maintaining high levels of school vaccine coverage.

## Notes

Supplement 1. **eTable 1.** Completeness of School-Level Immunization Data in the Study Population (n = 3632 Schools) **eTable 2.** School Characteristics Among Eligible Schools by Study Inclusion Status **eTable 3.** Final Selected Binomial Generalized Estimating Equations (GEE) Model With an Identity Link Function and Results Estimating the Impact of 2019 New York State (NYS) Senate Bill 2994A on Required Immunization Completion **eTable 4.** Final Selected Binomial Generalized Estimating Equations (GEE) Model With an Identity Link Function and Results Estimating the Impact of 2019 New York State (NYS) Senate Bill 2994A on Medical Exemption Uptake **eTable 5.** Final Selected Binomial Generalized Estimating Equations (GEE) Model With an Identity Link Function and Results Estimating the Impact of 2019 New York State (NYS) Senate Bill 2994A on the Uptake of All Vaccine Exemptions

Supplement 2.
Data Sharing Statement
