# Supplementary material for: School Vaccine Coverage and Medical Exemption Uptake After the New York State Repeal of Nonmedical Vaccination Exemptions
Source: JAMA Netw Open. 2024 Feb 2;7(2):e2354710. doi: 10.1001/jamanetworkopen.2023.54710 (PMC10837748; doi:10.1001/jamanetworkopen.2023.54710)
Supplement: Supplement 1. — eTable 1. Completeness of School-Level Immunization Data in the Study Population (n = 3632 Schools) eTable 2. School Characteristics Among Eligible Schools by Study Inclusion Status eTable 3. Final Selected Binomial Generalized Estimating Equations (GEE) Model With an Identity Link Function and Results Estimating the Impact of 2019 New York State (NYS) Senate Bill 2994A on Required Immunization Completion eTable 4. Final Selected Binomial Generalized Estimating Equations (GEE) Model With an Identity Link Function and Results Estimating the Impact of 2019 New York State (NYS) Senate Bill 2994A on Medical Exemption Uptake eTable 5. Final Selected Binomial Generalized Estimating Equations (GEE) Model With an Identity Link Function and Results Estimating the Impact of 2019 New York State (NYS) Senate Bill 2994A on the Uptake of All Vaccine Exemptions [file jamanetwopen-e2354710-s001.pdf]

Supplemental Online Content

Correira JW, Kamstra R, Zhu N, Doll MK. School vaccine coverage and medical exemption uptake after New York State repeal of nonmedical vaccination exemption. *JAMA Netw Open*. 2024;7(2):e2354710. doi:10.1001/jamanetworkopen.2023.54710

eTable 1. Completeness of School-Level Immunization Data in the Study Population (n=3,632 Schools)

eTable 2. School Characteristics Among Eligible Schools by Study Inclusion Status

eTable 3. Final Selected Binomial Generalized Estimating Equations (GEE) Model With an Identity Link Function and Results Estimating the Impact of 2019 New York State (NYS) Senate Bill 2994A on Required Immunization Completion

eTable 4. Final Selected Binomial Generalized Estimating Equations (GEE) Model With an Identity Link Function and Results Estimating the Impact of 2019 New York State (NYS) Senate Bill 2994A on Medical Exemption Uptake

eTable 5. Final Selected Binomial Generalized Estimating Equations (GEE) Model With an Identity Link Function and Results Estimating the Impact of 2019 New York State (NYS) Senate Bill 2994A on the Uptake of All Vaccine Exemptions

This supplemental material has been provided by the authors to give readers additional information about their work.

© 2024 Correira JW et al. *JAMA Network Open*

eTable 1. Completeness of school-level data in the study population (n=3,632 schools).

**# Years in Cohort**<br><br>(*i.e.*, # years eligible to submit immunization data)	**Included schools**<br><br># (%)	**Included schools with complete[1] immunization data**<br><br># (%)	**Included schools missing 1 year[2] of immunization data**<br><br># (%)	**Included schools missing ≥2 years[3] of immunization data**<br><br># (%)
10	3,126 (86.1)	3,061 (97.9)	49 (1.6)	16 (0.5)
9	201 (5.5)	192 (95.5)	8 (4.0)	1 (0.5)
8	111 (3.1)	98 (88.3)	7 (6.3)	6 (5.4)
7	28 (0.8)	25 (89.3)	1 (3.6)	2 (7.1)
6	59 (1.6)	53 (89.8)	5 (8.5)	1 (1.7)
5	44 (1.2)	39 (88.6)	3 (6.8)	2 (4.5)
4	43 (1.2)	39 (90.7)	4 (9.3)	0 (0.0)
3	12 (0.3)	10 (83.3)	2 (16.7)	0 (0.0)
2	8 (0.2)	8 (100)	0 (0.0)	0 (0.0)
**Total**	**3,632 (100)**	**3,525 (97.1)**	**79 (2.2)**	**28 (0.8)**

[1]*Complete immunization data*: schools where the # years with immunization data = # years in cohort

[2]*Missing 1 year*: schools where the # years with immunization data = (# years in cohort – 1)

[3]*Missing ≥2 years*: schools where the # years with immunization data = (# years in cohort – ≥2)

© 2024 Correira JW et al. *JAMA Network Open*

eTable 2. School characteristics among eligible schools by study inclusion status.

Characteristic	Included Schools (n = 3,632)	Excluded Schools (n = 189)
**Public Schools, %**	76.9 (95% CI: 75.6, 78.3)	46.6 (95% CI: 39.4, 53.7)
**# Schools with Demographic Data (2019)**	3,602	187
Mean Enrollment	457.3 (95% CI: 445.0, 469.6)	264.7 (95% CI: 216.7, 312.8)
White, %	65.8 (95% CI: 64.8, 66.8)	78.7 (95% CI: 74.8, 82.6)

© 2024 Correira JW et al. *JAMA Network Open*

eTable 3. Final selected binomial generalized estimating equations (GEE) model with an identity link function and results estimating the impact of 2019 New York State (NYS) Senate Bill 2994A on required immunization completion.

Final selected model:

$$Complete\ vx_{ij} = \beta_0 + \beta_1 Time_{ij} + \beta_2 School\ type_i + \beta_3 Time_{ij} * School\ type_i + \beta_4 Law_{ij} + \beta_5 Law_{ij} * School\ type_i + \beta_6 Time\ post\ law_{ij} + \beta_7 Time\ post\ law_{ij} * School\ type_i + \epsilon_{ij}$$

**Term**	**Estimate**	**P value**	**Lower CI**	**Upper CI**
Intercept	0.9855	<0.001	0.9844	0.9866
Time	-0.0017	<0.001	-0.0019	-0.0015
Law	0.0090	<0.001	0.0073	0.0106
Time post law	0.0027	<0.001	0.0017	0.0036
School type	-0.1134	<0.001	-0.1299	-0.0970
Time∗School type	-0.0034	<0.001	-0.0051	-0.0017
Law∗School type	0.0462	<0.001	0.0356	0.0567
Time post law∗School type	0.0073	0.0013	0.0028	0.0118

Where:

- $Complete\ vx_{ij}$ = Mean school required vaccine completion (%)
- $Time_{ij}$ = Time from the start of study period (years)
- Law_{ij} = NYS Senate Bill 2994A implementation (0=pre-implementation; 1=post-implementation)
- $Time\ post\ law_{ij}$ = Time from Senate Bill 2994A implementation (school years)
- $School\ type_{ij}$ = Represents public or nonpublic school (0=public or 1=nonpublic)
- ϵ = Error term
- i = Individual school
- j = Time

© 2024 Correira JW et al. *JAMA Network Open*

eTable 4. Final selected binomial generalized estimating equations (GEE) model with an identity link function and results estimating the impact of 2019 New York State (NYS) Senate Bill 2994A on medical exemption uptake.

$$Medical\ exemption\ uptake_{ij} = \beta_0 + \beta_1 Time_{ij} + \beta_2 School\ type_i + \beta_4 Law_{ij} + \beta_6 Time\ post\ law_{ij} + \epsilon_{ij}$$

**Term**	**Estimate**	**P value**	**Lower CI**	**Upper CI**
Intercept	0.0015	<0.001	0.0014	0.0017
Time	0.0001	<0.001	0.0000	0.0002
Law	-0.0006	0.0030	-0.0010	-0.0002
Time post law	-0.0002	<0.001	-0.0003	-0.0001
School type	0.0020	<0.001	0.0011	0.0029

Where:

- $Medical\ exemption\ uptake_{ij}$ = Mean school medical exemption uptake (%)
- $Time_{ij}$ = Time from the start of study period (years)
- Law_{ij} = NYS Senate Bill 2994A implementation (0=pre-implementation; 1=post-implementation)
- $Time\ post\ law_{ij}$ = Time from Senate Bill 2994A implementation (years)
- $School\ type_{ij}$ = Represents public or nonpublic school (0=public or 1=nonpublic)
- ϵ = Error term
- i = Individual school
- j = Time

© 2024 Correira JW et al. *JAMA Network Open*

eTable 5. Final selected binomial generalized estimating equations (GEE) model with an identity link function and results estimating the impact of 2019 New York State (NYS) Senate Bill 2994A on the uptake of all vaccine exemptions.

$$Overall\ exemption\ uptake_{ij} = \beta_0 + \beta_1 Time_{ij} + \beta_2 School\ type_i + \beta_3 Time_{ij} * School\ type_i + \beta_4 Law_{ij} + \beta_5 Law_{ij} * School\ type_i + \beta_6 Time\ post\ law_{ij} + \beta_7 Time\ post\ law_{ij} * School\ type_i + \epsilon_{ij}$$

Term	Estimate	P value	Lower CI	Upper CI
Intercept	0.0063	<0.001	0.0059	0.0066
Time	0.0007	<0.001	0.0007	0.0008
Law	-0.0098	<0.001	-0.0103	-0.0093
Time post law	-0.0008	<0.001	-0.0009	-0.0007
School type	0.0746	<0.001	0.0598	0.0894
Time∗School type	0.0021	<0.001	0.0012	0.0030
Law∗School type	-0.0874	<0.001	-0.1021	-0.0727
Timepost law∗School type	-0.0017	0.0024	-0.0028	-0.0006

Where:

- *Overall exemption uptake$_{ij}$* = Mean school medical and religious exemption uptake (%)
- *Time$_{ij}$* = Time from the start of study period (years)
- *Law$_{ij}$* = Represents period before and after Senate Bill 2994A implementation (0 or 1)
- *Time post law$_{ij}$* = Time from Senate Bill 2994A implementation (years)
- *School type$_{ij}$* = Represents public or nonpublic school (0 or 1)
- ϵ = Error term
- *i* = Individual school
- *j* = Time

© 2024 Correira JW et al. *JAMA Network Open*
