# Supplementary material for: School Vaccine Coverage and Medical Exemption Uptake After the New York State Repeal of Nonmedical Vaccination Exemptions
Source: JAMA Netw Open. 2024 Feb 2;7(2):e2354710. doi: 10.1001/jamanetworkopen.2023.54710 (PMC10837748; doi:10.1001/jamanetworkopen.2023.54710)
Supplement: Supplement 2. — Data Sharing Statement [file jamanetwopen-e2354710-s002.pdf]

Data Sharing Statement

Correira. School Vaccine Coverage and Medical Exemption Uptake After New York State Repeal of Nonmedical Vaccination Exemption. *JAMA Netw Open*. Published February 02, 2024. doi:10.1001/jamanetworkopen.2023.54710

Data
Data available: No

Additional Information
Explanation for why data not available: The data used in these analyses are publicly available from the New York State Department of Health and the New York State Education Department.
